# Chromosome numbers of *Carex* (Cyperaceae) and their taxonomic implications

**DOI:** 10.1371/journal.pone.0228353

**Published:** 2020-02-10

**Authors:** Helena Więcław, Anna Kalinka, Jacob Koopman

**Affiliations:** 1 Institute of Marine and Environmental Sciences, University of Szczecin, Adama Mickiewicza, Szczecin, Poland; 2 Institute of Biology, University of Szczecin, Wąska, Szczecin, Poland; 3 Molecular Biology and Biotechnology Center, University of Szczecin, Wąska, Szczecin, Poland; 4 ul. Kochanowskiego 27, Choszczno, Poland; The National Orchid Conservation Center of China; The Orchid Conservation & Research Center of Shenzhen, CHINA

## Abstract

Counting chromosomes is the first step towards a better understanding of the karyotype evolution and the role of chromosome evolution in species diversification within *Carex*; however, the chromosome count is not known yet for numerous sedges. In this paper chromosome counts were performed for 23 *Carex* taxa from Armenia, Austria, the Czech Republic, and Poland. Chromosome numbers were determined for the first time in three species (*Carex cilicica*, 2n = 54; *C*. *phyllostachys*, 2n = 56; *C*. *randalpina*, 2n = 78), two subspecies (*C*. *muricata* subsp. *ashokae*, 2n = 58; *C*. *nigra* subsp. *transcaucasica*, 2n = 84) and two hybrids (*C*. ×*decolorans*, 2n = 74; *C*. ×*walasii*, 2n = 108). Among the taxa whose number of chromosomes had been known before, the largest difference was found in *C*. *hartmaniorum* (here 2n = 52) and *C*. *aterrima* subsp. *medwedewii* (here 2n = 52). A difference in the chromosome count was demonstrated for *C*. *cilicica* (2n = 54) versus the species of the section *Aulocystis* (2n = 30 to 40) and for *C*. *tomentosa* (2n = 48) versus the species of the section *Acrocystis* (2n = 18 to 38). The results of this study indicate that the position of *C*. *cilicica* in *Aulocystis* section may raise doubts. Attention was paid to the relationship between *C*. *phyllostachys* and taxa of the subgenus *Carex* section *Gynobasidae*.

## Introduction

With about 2000 species described worldwide *Carex* L. (Cyperaceae) represents one of the most species-rich angiosperm genera [1]. The taxonomic richness is accompanied by an extreme variability in the number of chromosomes [2]. Sedges have holocentric chromosomes, which–in theory–guarantee a rapid karyotype evolution [3,4]. If a monocentric chromosome is fragmented, fragments lacking the centromere cannot be normally segregated during meiosis, which results in a loss of genetic material, the gametes produced being potentially non-viable [3]. The situation is different with holocentric chromosomes, because chromosome fragments are not lost, and a change in the chromosome count can be offset by, e.g., self-pollination or back-crossing. In addition, associations between non-homologous chromosomes during meiosis frequently do not disturb segregation, thus reducing selective pressure against chromosome rearrangements [5,6]. Although not all organisms with holocentric chromosomes show highly variable chromosome counts [7,8], the genus *Carex* is an ideal object to study the chromosome number variability [3,9]. The chromosome number variability in different species within a genus with monokinetic chromosomes is usually a result of polyploidy or aneuploidy [6,10,11]. In those species with holokinetic chromosomes, the frequent aneuploidy is complemented by two additional mechanisms which may lead to differences in the chromosome count: agmatoploidy (fission of chromosomes) and symploidy (fusion of chromosomes) [10,12–15]. It seems that evolution of karyotypes in sedges, important for species diversification, is driven by fusion and fission of chromosomes [16]. On the other hand, polyploidy is most likely rare in *Carex* [11,17].

The number of chromosomes in *Carex* varies from n = 6 to n = 62 and spans actually a continuous series from n = 6 to n = 47, more than 100 species showing different cytotypes [2,17]. In addition, the chromosome count is not known yet for numerous sedges [2,18]. Counting chromosomes is the first step towards a better understanding of the karyotype evolution and the role of chromosome evolution in species diversification within *Carex*. Therefore, the present work was aimed at: (i) analysing the chromosome counts in 23 *Carex* taxa, including 7 with hitherto unknown chromosome numbers, and (ii) exploring relationships between the number of chromosomes and taxonomy of the genus *Carex*.

## Materials and methods

### Plant material and specimen collection

Plants were collected in the field over the period 2013 to 2018 in Armenia, Austria, the Czech Republic, and Poland. Fieldwork was conducted outside protected areas, on sites where sedges were abundant. The study did not concern any protected taxa with the exception of *Carex secalina* in the Czech Republic. In this case, we took ripe utricles in the field, without harming the plant (Law No.114/1992 Coll., as amended 2 March 2008, On Protection of Nature and Landscape). The seeds were subjected to germination under greenhouse conditions. The seedlings were raised for about 1–2 months. Then, roots from the seedling were collected and used for studying the number of chromosomes.

Overall, the chromosome count was determined in 17 species, 3 subspecies, and 3 hybrids from 14 sections (Table 1). For taxonomic identification some experts were consulted: A. Molina–section *Phaestoglochin* Dumort., R. Řepka–section *Ammoglochin* Dumort. and section *Phaestoglochin*, and B. Wallnöfer–section *Phacocystis* Dumort. The specimens examined were compared with herbarium specimens kept in B, ERE, H, W (particularly with the type material of *C*. *randalpina* B.Walln., *C*. ×*oenensis* A.Neumann ex B.Walln., *C*. *muricata* subsp. *ashokae* Molina Gonz., Acedo & Llamas and *C*. *otomana* Molina Gonz., Acedo & Llamas at the herbarium of the Natural History Museum in Vienna, W*)*. In addition, specimens of *C*. *curvata* Knaf *and C*. ×*walasii* M.Ceynowa-Giełdon were collected from the *locus classicus* (see Table 1), while *C*. *cilicica* Boiss. was collected from the only site known in Armenia, which has been well-documented in the herbarium of ERE.

**Table 1:** Chromosome numbers of studied taxa against the background of available literature data. Taxa with chromosome numbers reported for the first time or with chromosome numbers significantly different from literature data are given in bold.

Taxon	2n	Subgenus/ Section	Literature data			Locality/date/collector	Distribution
			n	2n	References		
***Carex aterrima* subsp. *medwedewii***	**52**	*Carex/ Racemosae*		32	[25]	Armenia, Aragatsotn mars, S flank of Mt. Aragats, valley bottom of gorge W of road to Kari lake, alpine meadow, 40°26'54"N 44°11'51"E, 2810 m a.s.l./3 July 2015/leg. Więcław H.	Caucasus, Turkey, Iran, and Iraq [19]
*Carex bohemica*	80	*Vignea/ Cyperoideae*		80	[18,26,27,28]	Poland, Lubuskie Province, Milowice, dried up fish pond shore, 51°36ʹ04.6ʺ N 15°03ʹ23.7ʺ E/27 July 2013/leg. Więcław H.	Eurasian species; from western Europe to Japan [19,20]
			40		[29]		
				62–64	[30]		
				c. 60	[31]		
				c. 62	[32]		
*Carex buxbaumii*	102	*Carex/ Racemosae*		100	[18,33]	Poland, Western Pomerania, E of Gizyn, along Miedwie Lake, *Phragmitetum* along lake shore, 53°13'30.32"N 14°51'59"E/28 May 2013/leg. Więcław H.	Eurasia and N America [1,19,20]
				106	[34]		
				74	[35]		
***Carex cilicica***	**54**	*Carex/ Aulocystis*	**-**	**-**	**-**	Armenia, Vayots' Dzor mars, c. 14 km S of Yeghegnadzor, c. 3 km SE Gnishik, former road to Khachik, besides rivulet, 39°38'08"N 45°19'19"E, 2300 m a.s.l./08 July 2015/leg. Koopman J.	Armenia, Turkey, Iran and Iraq [19]
*Carex curvata*	58	*Vignea/ Ammoglochin*		58	[18,36]	Czech Republic, Bohemia, Doubi forest near Chomutov town, *locus classicus*; *Quercus petraea* agg.-forest, 50°27ʹ38.5ʺN 13°27ʹ94.3ʺ E/10 May 2014/leg. Więcław H.	Germany, the Czech Republic, Slovakia, Hungary, Romania, Austria, Switzerland, Poland, Belgium [48], and Ukraine (R. Řepka, pers. comm.)
**Carex ×decolorans**	**74**	*Carex/ Phacocystis*	**-**	**-**	**-**	Austria, Steiermark, S of Zirbitzkogel, along small rivulet in alpine meadow on silicate substrate, 47°03ʹ54.1ʺ N 14°35ʹ12.8ʺ E, 2088 m a.s.l./6 July 2014/ leg. Więcław H.	Eurpe, N America [20]
*Carex diluta*	74	*Carex/ Spirostachyae*		56	[37]	Armenia, Geghark'unik' mars, NE-side of lake Sevan, gorge NE of Pambak, besides rivulet, 40°23'13"N 45°32'09"E, 2025 m a.s.l./4 July 2015/leg. Koopman J. & Więcław H.	Caucasus and Middle Asia [19]
	74			70	[38]	Armenia, Geghark'unik' mars, NE-side of lake Sevan, at coast c. 5.3 km SE of Artanish, humid, partly boggy meadow and besides rivulet, 40°27'56"N 45°24'50"E, 1915 m a.s.l./4 July 2015/leg. Więcław H.	
***Carex hartmaniorum***	**52**	*Carex/ Racemosae*		68	[11]	Armenia, Geghark'unik' mars, c. 12 km SSW of Martuni, SW of small village, c. 0.25 km W of road to Selim pass, humid meadow with drier spots, 40°02'07"N 45°14'33"E; 2260 m s.m/7 July 2015/leg. Koopman J. & Więcław H.	C and E Europe and adjacent parts of Asia [19,20]
	**52**					Poland, Western Pomerania, Otanów, E of Jezioro Chłop, wet meadow, 52°59'21.41"N 14°54'0.11"E/28 May 2018/ leg. Koopman J.	
*Carex hordeistichos*	58	*Carex/ Secalinae*		54	[39]	Armenia, Yerevan mars, road Yerevan to Garni, NW of Voghjaberd, below Charents arch, meadow, 40°10'22"N, 44°38'07"E, 1600 m a.s.l./2 July 2015/leg. Koopman J.	Europe, Caucasus, Asia (Turkey, Iran, Iraq) and N Africa [19]
	58			54–60	[40]	Austria, N of Oed, along a path, open space in *Fagus*-forest with wild boar baths; calcareous soil, 47°53ʹ92.7ʺ N 16°02ʹ66.7ʺ, 762 m a.s.l./5 July 2014/leg. Więcław H.	
				56	[27,36,41]		
			28		[42]		
				58	[18]		
***Carex muricata* subsp. *ashokae***	**58**	*Vignea/ Phaestoglochin*	**-**	**-**	**-**	Armenia, Aragatsotn mars, S flank of Mt. Aragats, road to Hamberd, group of houses c. 4 km N of Bjurakan, shady semi-ruderal meadow, 40°22'31"N 44°16'00"E, 1965 m a.s.l./3 July 2015/ leg. Więcław H.	mountains of Eastern Europe and the Middle East, from the Caucasus and the Kars towards Central Asia, through the Zagros Mountains to the Pamirs and Targabatay [43]
	**58**					Armenia, Lorri mars, road Vanadsor–Stepanavan, between road turns 3.7 km S Gargar, meadow, 40°55'26"N 44°26'23"E, 1735 m a.s.l./5 July 2015/ leg. Więcław H.	
***Carex nigra* subsp. *transcaucasica***	**84**	*Carex/ Phacocystis*	**-**	**-**	**-**	Armenia, Geghark'unik' mars, c. 12 km SSW of Martuni, WSW of small village, c. 0.23 km W of road to Selim pass, 40°02'07"N 45°14'34"E, 2265 m a.s.l./7 July 2015/leg. Koopman J.	Caucasus (except Ciscaucasia) and Turkey [19]
*Carex* ×*oenensis*	84	*Carex/ Phacocystis*		±84	[44]	Austria, Niederösterreich, Voralpen, Ybbstal, near Lunzersee, Lunz am See, wet place along path, 47°51ʹ16.6ʺ N 15°03ʹ43.7ʺ E, 618 m a.s.l./7 July 2014/leg. Więcław H.	Germany, Austria, Italy and Slovenia [20]
*Carex otomana*	56	*Vignea/ Phaestoglochin*		54	[11]	Czech Republic, Bohemia, near Chomutow town, along forest path near the road, 50°27ʹ37.5ʺ N, 13°28ʹ05.4ʺ E/10 May 2014/leg. Więcław H.	from east of the Black Sea (Bulgaria) and Greece through the Turkish mountains and the Caucasus to the mountains on the west side of Tyan Shan in Central Asia (Kazakhstan) [45]
*Carex pairae*	58	*Vignea/ Phaestoglochin*	26	52	[46]	Poland, Zachodniopomorskie Province, Łowicz Wałecki, W of Mirosławiec, roadside along sand path, 53°20ʹ10.7ʺ N 16°02ʹ08.4ʺ E/6 August 2013/leg. Koopman J.	Europe, Azores, NW Africa [20]
				56	[47]		
			29		[29]		
				58	[28,48]		
*Carex pallidula*	56	*Carex/ Clandestinae*	27	54	[49]	Czech Republic, Bohemia, Rakovník District, the village of Milý, sunny, calcareous slope with *Orchis purpurea*, 50°14ʹ10.3ʺN 13°52ʹ47.4 ʺ E/ 11 May 2014/ leg. Więcław H.	N Europe and in central and southeastern parts of Europe, from the highlands in the south of Poland to the northern part of the Balkan Peninsula [51]
				c. 51	[50]		
***Carex phyllostachys***	**56**	*Psyllophora/ Caryotheca* (*Schoenoxiphium* clade)	**-**	**-**	**-**	Armenia, Syunik Province, area c. 9 km SE Kapan, road between Chakaten and Shikahogh, Steep slope along stream in *Quercus*-forest, 985 m a.s.l., 39°08'28" N 46°27'50" E/16 June 2016/leg. Więcław H.	S Europe (Italy, Macedonia, Albania, and Greece), the Caucasus and W Asia [19,20]
***Carex randalpina***	**78**	*Carex/ Phacocystis*	**-**	**-**	**-**	Austria, Voralpen, Ybbstal, Lunz am See, Lunzersee, along small ditch between road and meadow, 47°51ʹ15.9ʺN 15°04ʹ12.0ʺE, 619 m a.s.l./7 July 2014/leg. Więcław H.	Germany, Austria, Slovenia and Switzerland, northern Croatia, north-eastern Italy and Hungary [20]
*Carex repens*	70	*Vignea/ Ammoglochin*		70	[36]	Poland, Kujawsko-Pomorskie Province, E of Przyłubie, N of road no 10, *Pinus*-forest, on top of slope to Wisła, 53°2'54.13"N 18°22'22.48"E/12 July 2016/leg. Koopman J.	Austria, Hungary, Italy, Poland, and Romania [20]
*Carex secalina*	50	*Carex/ Secalinae*		50	[19]	Armenia, Geghark'unik' mars, 3 km SSW of Sevan, E of Lchashen, meadow between road and lake, 40°31'26"N 44°56'50"E, 1910 m a.s.l./7 July 2015/leg. Koopman J.	C Europe to C Asia [20]
	50					Czech Republic, Bohemia, NE of Louny, roadside 50°24ʹ05.3ʺ N, 13°57ʹ66.3ʺ/12 May 2014/leg. Koopman J. & Więcław H.	
*Carex songorica*	82	*Carex/ Tumidae*		82	[52]	Armenia, Geghark'unik' mars, road Sevan—Martuni, N of Lichk, meadow in former fish ponds, partly boggy, 40°10'11"N 45°14'26"E, 1925 m a.s.l./7 July 2015/leg. Więcław H.	Caucasus, Iran, Kazakhstan, Afghanistan, west Pakistan, S Siberia, Mongolia, and Turkey [19]
*Carex supina*	38	*Carex/ Lamprochlaenae*		38	[36]	Czech Republic, Bohemia, Holedeč, at the top of dry, steep silicate slope, 50°17ʹ04.7ʺ N 13°34ʹ12.3ʺ E/01 May 2014/leg. Więcław H.	C Europe, W Asia, boreal and subarctic N America [1,19]
*Carex tomentosa*	48	*Carex/ Acrocystis*		48	[28,53–55]	Armenia, Vayots' Dzor mars, c. 13 km S of Yeghegnadzor, c. 2.6 km SE Gnishik, former road to Khachik, meadow, 39°38'18"N 45°19'11"E, 2270 m a.s.l./8 July, 2015/leg. Więcław H.	Eurasian species with its eastern distribution limits in E Siberia and Mongolia; it also occurs in Turkey and N Iran [19,20]
			24		[29,56]		
***Carex* ×*walasii***	**108**	*Carex/ Carex*	**-**	**-**	**-**	Poland, Zachodniopomorskie Province, between Storkowo and Studnica, S of road, along shore of former, overgrown pond, 53°27'50.4"N 15°36'3.6"E/ 21 June 2014/ leg. Koopman J.	Poland and Germany [20]
	**108**					Poland, Kujawsko-Pomorskie Province, Łowinek *locus classicus*, SW point of pond, along hayland, 53°21ʹ43.4ʺ N 18°67ʹ70.7ʺ E/8 June 2014/leg. Koopman J.	

Voucher specimens for each taxon were deposited in the Herbarium Stetinensis at the University of Szczecin (SZUB). The nomenclature used follows Egorova [19] and Koopman [20], except for *C*. *curvata* [21], *C*. *hartmaniorum* A.Cajander [22], and *C*. *nigra* subsp. *transcaucasica* (T.V.Egorova) Jim.Mejías, G.E.Rodr.-Pal., Amini Rad & Martín-Bravo [23]. The names of sections used follow Egorova [19], Reznicek [24], and Ball & Reznicek [1].

### Chromosome counts

Plant cuttings were transferred from soil to hydroponic cultures. When the new roots emerged, they were excised and immersed in ice-cold water for 16 h. The roots were subsequently fixed in Carnoy’s solution (absolute ethanol: glacial acetic acid 3:1 v/v) for 24 hours at 4°C. They were carefully washed in distilled water, and the root tips were dissected. Each root tip was macerated directly on a microscope slide in a mixture of 4% (w/v) pectinase (Fluka, Buchs, Switzerland), 6% (w/v) hemicellulase (Sigma-Aldrich, St. Louis, USA) and 4% (w/v) cellulase (Sigma-Aldrich, St. Louis, USA) in 0.01 M citric acid-sodium citrate buffer (pH 4.8), for 5 hours at 37°C in a humidity chamber. Root tips were washed with 0.01 M citric acid-sodium citrate buffer (pH 4.8) and then with 45% acetic acid. Root tips were squashed under a cover glass. The cover slip was removed after freezing over dry ice, and the slides were air-dried overnight. The slides were dehydrated in a graded ethanol series (70%, 96%, and 99.8%) at room temperature, air-dried and stained with DAPI (1 μg/mL) (Sigma-Aldrich, St. Louis, USA) for 15 min. The slides were rinsed 3× with distilled water, air-dried and mounted in Vectashield® Hard Set mounting medium for fluorescence (Vector Laboratories, Burlingame, USA) and analysed with the Axio Imager Z2 epifluorescence microscope (Carl Zeiss, Oberkochen, Germany). The resultant images were captured and analysed using the GenASIs software (Applied Spectral Imaging). About 60 slides per taxon were prepared and analysed (2 preparations × 30 plants per taxon). The accurate counting was carried out in at least 60 metaphase spreads per each taxon.

## Results

**Fig 1:**
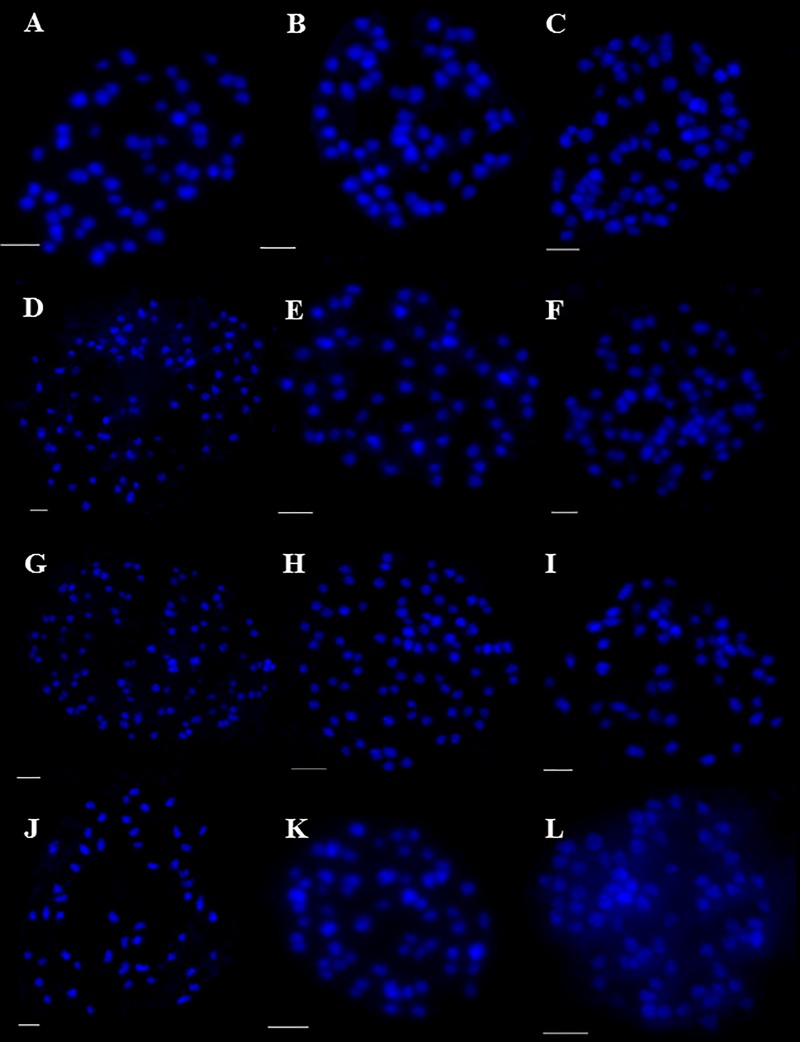
Mitotic metaphase chromosome spreads of the analysed *Carex* taxa. (A) *C*. *cilicica*, 2n = 54; (B) *C*. *phyllostachys*, 2n = 56; (C) *C*. *randalpina*, 2n = 78; (D) *C*. *nigra* subsp. *transcaucasica*, 2n = 84; (E) *C*. *muricata* subsp. *ashokae*, 2n = 58; (F) *C*. ×*decolorans*, 2n = 74; (G) *C*. ×*walasii*, 2n = 108; (H) *C*. ×*oenensis*, 2n = 84; (I) *C*. *hartmaniorum*, 2n = 52; (J) *C*. *aterrima* subsp. *medwedewii*, 2n = 52; (K) *C*. *curvata*, 2n = 58; (L) *C*. *repens*, 2n = 70.

This paper is the first to provide chromosome numbers for seven *Carex* taxa belonging to five sections (Table 1). This applies to three species: *C*. *cilicica* (2n = 54, section *Aulocystis* Dumort.; Fig 1A), *C*. *phyllostachys* C.A.Mey. (2n = 56, sect. *Caryotheca* V.Krecz. ex Egor.; Fig 1B) and *C*. *randalpina* (2n = 78, sect. *Phacocystis*.; Fig 1C), two subspecies: *C*. *nigra* subsp. *transcaucasica* (2n = 84, sect. *Phacocystis*; Fig 1D) and *C*. *muricata* subsp. *ashokae* (2n = 58, sect. *Phaestoglochin*; Fig 1E) and two hybrids: *C*. ×*decolorans* Wimm. (2n = 74, *C*. *bigelowii* Torr. ex Schwein. × *C*. *nigra* (L.) Reichard, sect. *Phacocystis*; Fig 1F) and *C*. ×*walasii* (2n = 108, *C*. *atherodes* Spreng. × *C*. *hirta* L., sect. *Carex*; Fig 1G). In hybrids, *C*. ×*decolorans* and *C*. ×*walasii*, we observed an intermediate chromosome numbers between those of the putative parents, while the chromosome count in *C*. ×*oenensis* (2n = 84, *C*. *acuta* L. × *C*. *randalpina*; Fig 1H) was almost identical to that in *C*. *acuta* (see Discussion).

**Fig 2:**
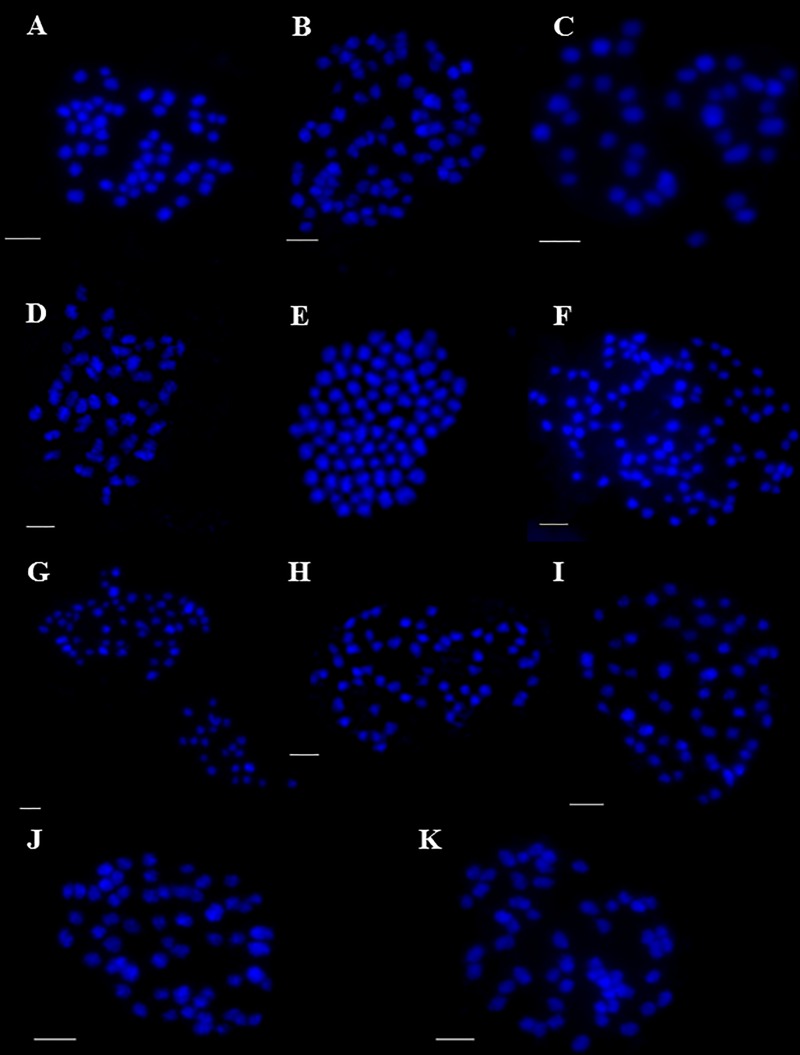
Mitotic metaphase chromosome spreads of the analysed *Carex* taxa. (A) *C*. *secalina*, 2n = 50; (B) *C*. *songorica*, 2n = 82; (C) *C*. *supina*, 2n = 38; (D) *C*. *tomentosa*, 2n = 48; (E) *C*. *bohemica*, 2n = 80; (F) *C*. *buxbaumii*, 2n = 102; (G) *C*. *diluta*, 2n = 74; (H) *C*. *hordeistichos*, 2n = 58; (I) *C*. *otomana*, 2n = 56; (J) *C*. *pairae*, 2n = 58; (K) *C*. *pallidula*, 2n = 56. Scale bar = 2.5 μM.

Comparison with the taxa whose chromosome numbers had been reported by other authors revealed the largest differences in *Carex hartmaniorum* (2n = 52; Fig 1I) and in *C*. *aterrima* subsp. *medwedewii* (Lesk.) T.V. Egorova (2n = 52; Fig 1J) (see Discussion and Table 1). On the other hand, in some species the number of chromosomes was consistent with the literature data: *Carex curvata* (2n = 58; Fig 1K), *Carex repens* Bellardi (2n = 70; Fig 1L), *Carex secalina* Wahlenb. (2n = 50; Fig 2A), *C*. *songorica* Kar. & Kir. (2n = 82; Fig 2B), *C*. *supina* Wahlenb. (2n = 38; Fig 2C) and *C*. *tomentosa* L. (2n = 48; Fig 2D). In the following species: *Carex bohemica* Schreb. (2n = 80; Fig 2E), *Carex buxbaumii* Wahlenb. (2n = 102; Fig 2F), *Carex diluta* M.Bieb. (2n = 74; Fig 2G), *Carex hordeistichos* Vill. (2n = 58; Fig 2H), *Carex otomana* http://www.ipni.org/ipni/idPlantNameSearch.do?id=77088652-1&back_page=%2Fipni%2FeditSimplePlantNameSearch.do%3Ffind_wholeName%3DCarex%2Botomana%26output_format%3Dnormal (2n = 56; Fig 2I), *Carex pairae* F.W.Schultz (2n = 58; Fig 2J), *Carex pallidula* Harmaja (2n = 56; Fig 2K) there were smaller or larger discrepancies in chromosome numbers in relation to previous data (see Discussion and Table 1).

Chromosomes of all *Carex* species are very small making it impossible either karyotyping or determining the presence of structural aberration. Their identification based upon morphological features and size is unreliable. We have measured the chromosome lengths in 25 randomly chosen metaphase spreads of different species. The mean length of a *Carex* chromosome, based on 1600 measurements, was 1.01 μM (σ = 0.27) with minimum 0.48 μM and maximum 1.92 μM. It is because of their size that the analysis of the number of chromosomes was carried out in as many as 60 metaphase plates from each taxon. Only in this way the error can be avoided and the results are authenticated.

## Discussion

### Chromosome numbers

The records, 2n = 32 for *Carex aterrima* subsp. *medwedewii*, cited by Gvinianidze & Avazneli [25], and 2n = 68 for *C*. *hartmaniorum*, reported by Lipnerová et al. [11] are doubtful because a similar chromosome number has not been recorded within the section *Racemosae* G. Don to which these taxa belong. Generally, within this section, two groups of cytotypes are given: the first group with 2n between 50–60 and the second with 2n between 100–106 [2]. Lipnerová et al. [11] addresses the section *Racemosae* as the product of polyploidy. Identification of polyploids in *Carex* is extremely difficult. In the case of autopolyploidy, a tetraploid species is expected to have twice as many chromosomes (in this work: *C*. *buxbaumii*, 2n = 102 and *C*. *hartmaniorum*, 2n = 52) and twice as big a genome than the initial diploid species. However, should the polyploidy event be relatively ancient evolutionarily, this direct relationship is most often blurred by a DNA sequence loss/acquisition, aneupolyploidy etc. occurring during evolution [57]. That is why different evolutionary scenarios in case of *Carex aterrima* subsp. *medwedewii* and *C*. *hartmaniorum* are possible. It can be hypothesised, that among *Carex aterrima* subsp. *medwedewii* there exist a diploid form (2n = 32) and a polyploidy one, which during its evolution has undergone different aneuploidy events, reaching the chromosome number of 52. It is confirmed by many studies that in neopoliploids a “genomic shock” occurs, which leads to many dysploidy and aneuploidy [58]. These changes are often inevitable to make the polyploid genome stable, properly functioning. Moreover, because *Carex* chromosomes are holocentric it can be expected that aneuploidy may occur on a larger scale. Therefore, it cannot be excluded, that large discrepancies in the number of chromosomes exist in one species, like for example in *C*. *hartmaniorum* (2n = 52 in this work, 2n = 68 in [11]).

The chromosome numbers in the remaining taxa examined in this work proved identical with or similar to literature data. Although the somatic chromosome number in *C*. *bohemica* was reported to be about 60 [30–32], other authors [18,26–28] provided data indicating the chromosome count to be identical with that found in this work (2n = 80). However, as stated above, parallel existence of different cytotypes, even with a very diverse number of chromosomes is possible.

The difference in the chromosome counts, between this study and data reported in the literature, for *C*. *buxbaumii*, *C*. *diluta*, *C*. *hordeistichos*, *C*. *otomana*, *C*. *pairae* and *C*. *pallidula* could have resulted from a number of reasons. The first involves the technical difficulty of counting the very small chromosomes, whereby some authors report their counts as approximate, using „±” or „ca.”. The *Carex* chromosomes are indeed small (ca. 1μm), which greatly hinders accurate counting; the relatively high number of chromosomes is an additional difficulty. This is, however, not the reason with which to plausibly explain such large discrepancies in the chromosome numbers in *C*. *hartmaniorum* and *C*. *aterrima* subsp. *medwedewii*. Another possible explanation of the discrepancy is a potential species misidentification. The third reason, probably the most important one, is the between-populations [59,60] or even between-individuals [61,62] variability. In addition, some species show a correlation between distribution at certain latitudes and the chromosome count variation [17,59,63]. However, the latitude-chromosome number correlation is not direct, and there is no pattern indicating an increase or a reduction in the chromosome number with latitude [62,64]. As we were comparing the chromosome numbers between sedges collected in Armenia and Poland (*C*. *hartmaniorum*) as well as in Armenia and the Czech Republic (*C*. *secalina*), we found no between-populations differences.

Taking into account the difficulties in determining the chromosome number, the comparison of our results with the literature data [2,18,36,52] indicates that a relatively stable chromosome number can be regarded as most likely in *C*. *curvata* (2n = 58), *C*. *secalina* (2n = 50), *C*. *songorica* (2n = 82), *C*. *supina* (2n = 38), *C*. *repens* (2n = 70), and *C*. *tomentosa* (2n = 48).

According to Cayouette & Morisset [65] and Cayouette & Catling [66], the chromosome numbers of hybrids were usually intermediate between those of the putative parents or equal to one of the parents if they differ only by one or two chromosomes. *Carex* ×*decolorans* had intermediate chromosome number between *C*. *bigelowii*, 2n = 68–70 and *C*. *nigra*, 2n = 80–86. In addition, an intermediate number of chromosomes was observed in *C*. ×*walasii* (*C*. *atherodes*, 2n = 74 and *C*. *hirta*, 2n = 112–114); the chromosome count in *C*. ×*oenensis* was very close to that in *C*. *acuta* (2n = 82–86) [2,18,34,62,67].

### Relationship between chromosome numbers and taxonomy of the genus *Carex*

**Fig 3:**
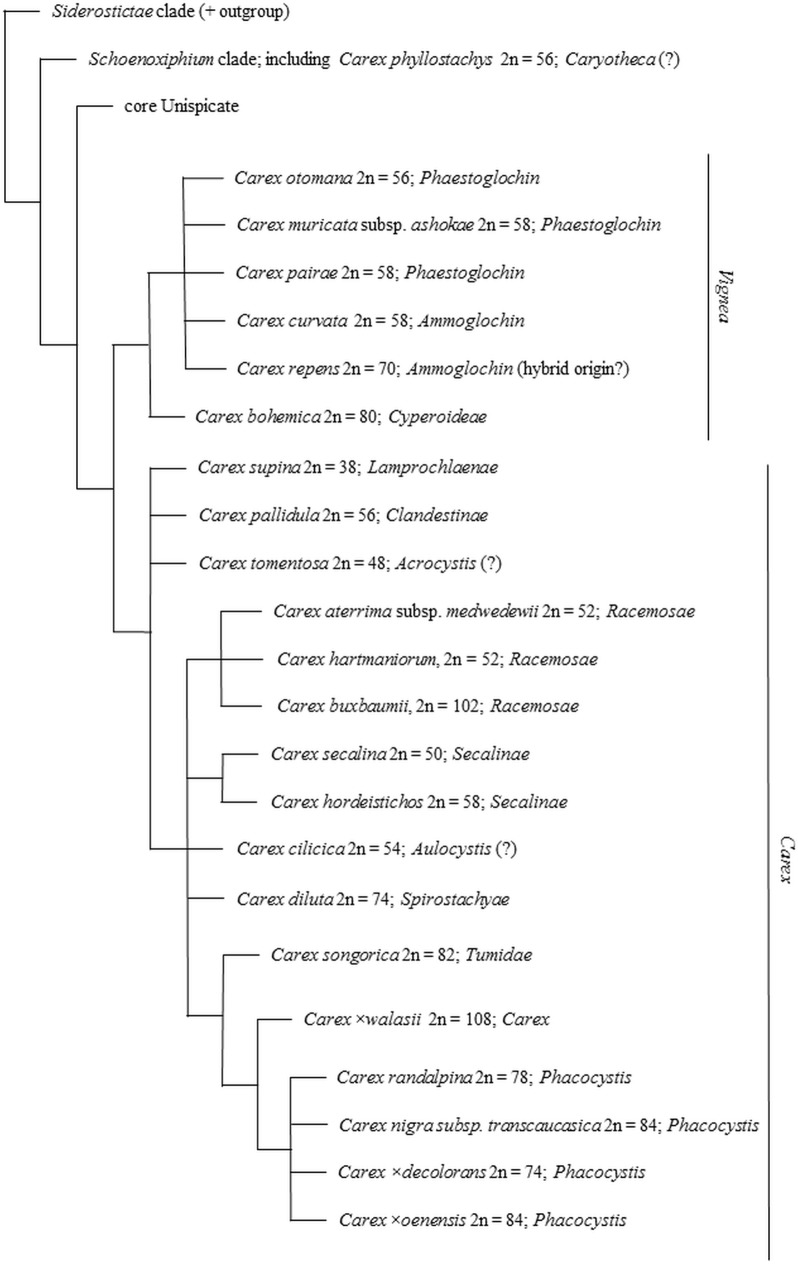
Schematic dendrogram based on the phylogenetic tree suggested by Global *Carex* Group [70] showing chromosome number variation against the background of phylogenetic relationships among studied taxa.

The genus *Carex* seldom shows discontinuities in the chromosome count series at the intraspecific level or in species aggregates; discontinuities, however, do usually occur between sections or subsections [2,68]. This is in line with the scenario whereby sedge species gradually accumulate chromosome rearrangements, which is reflected in the selection dynamics at the cellular level or in non-random cytotype extinction, and generates discontinuities usually observed at the level of section or subsection [68]. However, the subgenus *Vignea* frequently shows similar (or even identical) chromosome counts at the section level, e.g. sections *Ammoglochin* and *Phaestoglochin* both have the dominant cytotype 2n = 58 [2,18]. In this case, the numbers of chromosomes are hardly suitable for species identification, e.g. *C*. *brizoides* L., *C*. *curvata* and *C*. *praecox* Schreb. (section *Ammoglochin* Dumort.) as well as *C*. *muricata* subsp. *ashokae*, *C*. *pairae* and *C*. *divulsa* Stokes (section *Phaestoglochin*). Within the section *Ammoglochin*, a clearly different chromosome number occurs in *C*. *repens* (2n = 70), most probably of hybrid origin [69]. The subgenus *Vignea* is regarded as monophyletic, whereas the remaining subgenera established earlier (*Carex*, *Indocarex* and *Psyllophora*) are considered polyphyletic [70]. Results of recent phylogenetic studies showed the genus *Carex* to encompass five groups: the *Siderostictae* clade, the *Schoenoxiphium* clade, the core Unispicate, *Vignea* and the core Carex [70; see also Fig 3].

The chromosome numbers in the subgenus *Carex* (core Carex) are usually different at section level and may be useful for establishing the status of a taxon in the sedge classification system. Chromosome numbers in the section *Aulocystis* usually range within 2n = 30–40, but the section contains also species with the cytotype 2n = 54 (*C*. *cilicica*; this study) or 2n = 56 and 58 (*C*. *frigida* All.; see [2]). This section, divided into numerous subsections [19], proves to be polyphyletic [70]. Some taxa, e.g. *Carex frigida* mentioned above, are–on the phylogenetic tree–far removed from the remaining members of the section *Aulocystis* [70,71]. Similarly, the taxonomic status of *C*. *cilicica* is not clear. Owing to differences in morphology [19] and the chromosome number between *C*. *cilicica* and taxa of the section *Aulocystis*, it seems hardly likely that the sedge is closely related to them. Kükenthal [72] assigned this species to the subsection *Fuliginosae* Tuckerm. within the section *Frigidae* Fries. (= *Aulocystis*), whereas Nilsson [73] put it in the section *Fulvellae* Fries ex Christ. The latter has been recently divided into two closely related sections *Spirostachyae* Drej. ex L.H. Bailey and *Ceratocystis* Dumort. [19,74]. The chromosome numbers in the section *Spirostachyae* are relatively well known and a substantial cytogenetic variability, 2n = 60–84, has been found [38,59,68,75,76]. The chromosome numbers in the section *Ceratocystis* range within 2n = 56–72 [61,77,78]. Most probably, the inclusion of *C*. *cilicica* in the section *Ceratocystis* or *Spirostachyae* rather than in the section *Aulocystis* would be more appropriate; therefore further studies–molecular analyses in particular–are necessary for unequivocal resolution of the taxonomic position of this species within the subgenus *Carex*.

The chromosome numbers in the section *Acrocystis* Dumort. usually range from 2n = 18 to 2n = 38 [2]. In this study, *C*. *tomentosa* was confirmed to belong to a cytotype of 2n = 48 which seems to be stable in this species [18]. According to Roalson, et al. [79], the section *Acrocystis* appears to be polyphyletic and some species, e.g. *C*. *grioletii* Roem. and *C*. *tomentosa*, should be excluded from it. This seems justified also because of differences in the chromosome numbers (2n = 48 in *C*. *grioletii* [80]). Kükenthal [72] included these species in the section *Pachystylae* Kükenth., whereas Egorova [19] assigned them to different subsections (the *Elongatibracteatae* Egor. and the *Tomentosae* Egor.) within the section *Acrocystis*. Phylogenetic studies carried out by the Gobal *Carex* Group [70] showed the species to be located at different sites on the phylogenetic tree: *C*. *grioletii* was within the section *Thuringiaca* G. Don., while *C*. *tomentosa* was placed in the vicinity of the section *Paniceae* G. Don [71]. In our opinion, the position of these species in the sedge classification system is not clear and requires further study.

The chromosome number in the *Clandestinae* G. Don is usually 2n = 35–56, except for *C*. *callitrichos* V.I.Krecz., *C*. *lanceolata* Boott and *C*. *rhizina* Blytt ex Lindblom which are all polyploid with 2n = 70 cytotype [11,18]. The section *Clandestinae* is a large and inhomogeneous group which is divided into numerous subsections [19]. Some taxa resemble one another morphologically and have similar distribution, e.g., *C*. *digitata* L. and *C*. *pallidula*, which renders their identification difficult [51]. Perhaps the chromosome numbers will prove useful in the identification of those species. The cytotype of *Carex pallidula* is 2n = 56 (as reported here) or 2n = 54 [49], whereas 2n = 52 appears to be the most frequent chromosome number in *C*. *digitata* throughout the whole natural range of the species [2]. Although Roalson [2] reported a cytotype variation (2n = 48, 2n = 50, 2n = 52, 2n = 54, and 2n = 56) in the latter taxon, the variation could have been caused by the fact that *C*. *digitata* s.l. has been split up recently in *C*. *digitata* s.s. and *C*. *pallidula* [81,82]. However, more detailed studies covering other areas of their occurrence are necessary to confirm that the number of chromosomes is appropriate for distinguishing these species.

The chromosome numbers in the remaining sections within the subgenus *Carex* studied here, *Lamprochlaenae* (Drejer) L. H. Bailey, *Phacocystis*, *Tumidae* Meinsh. and *Secalinae* (O.Lang) Kük. did not deviate from those reported in literature, 2n = 34–38, 2n = 60–88, 2n = 70–80, and 2n = 50–60, respectively [2,18,39,67].

Recent phylogenetic studies have demonstrated a close relationship between *C*. *phyllostachys* and the sedges of the subgenus *Carex* section *Gynobasidae* Trabut.: *C*. *illegitima* Ces. and *C*. *oedipostyla* Duval-Jouve within *Schoenoxiphium* clade [70; see also Fig 3], but this taxa substantially differ in morphology [74]. Most likely, *C*. *phyllostachys* is not closely related to the section *Phyllostachyae* Tuckerman ex Kükenthal species [70], the section grouping species occurring in North America [83]. The *Phyllostachyae* species’ chromosome numbers range from 2n = 62 to 2n = 98 [84], the chromosome count in *C*. *phyllostachys* being 2n = 56 (determined in this study). The chromosome numbers in *C*. *illegitima* and *C*. *oedipostyla* are not known yet. Information on the chromosome counts in those species will most likely help to gain insight into the relationship between them and *C*. *phyllostachys*, because, as observed by Heilborn [35], closely related carices frequently show similar numbers of chromosomes.
